# The prediction of Metabolic Syndrome alterations is improved by combining waist circumference and handgrip strength measurements compared to either alone

**DOI:** 10.1186/s12933-021-01256-z

**Published:** 2021-03-22

**Authors:** Jose P. Lopez-Lopez, Daniel D. Cohen, Daniela Ney-Salazar, Daniel Martinez, Johanna Otero, Diego Gomez-Arbelaez, Paul A. Camacho, Gregorio Sanchez-Vallejo, Edgar Arcos, Claudia Narvaez, Henry Garcia, Maritza Perez, Dora I. Molina, Carlos Cure, Aristides Sotomayor, Álvaro Rico, Eric Hernandez-Triana, Myriam Duran, Fresia Cotes, Darryl P. Leong, Sumathy Rangarajan, Salim Yusuf, Patricio Lopez-Jaramillo

**Affiliations:** 1 Institute Masira, Medical School, Universidad de Santander, Santander, Colombia; 2 Facultad de Ciencias de La Salud, Instituto de Investigaciones Masira, Universidad de Santander (UDES), Bloque G, piso 6. Bucaramanga, Santander, Colombia; 3 Universidad del Quindío and Hospital San Juan de Dios de Armenia, Armenia, Quindío, Colombia; 4 Fundación Cometa, Pasto, Colombia; 5 Hospital Susana López, Popayán, Colombia; 6 Fundación RIESCAR, El Espinal, Colombia; 7 Facultad de Medicina, Universidad Militar Nueva Granada, Bogotá, Colombia; 8 Universidad de Caldas y Médicos Internistas de Caldas, Manizales, Colombia; 9 BIOMELAB Research Center, Barranquilla, Colombia; 10 Centro Cardiovascular Santa Lucia, Cartagena, Colombia; 11 FINDEMOS, Yopal, Colombia; 12 ENDOCARE, Bogotá, Colombia; 13 Universidad de Santander, Valledupar, Colombia; 14 PHRI, McMaster University, Hamilton, ON Canada

**Keywords:** Metabolic syndrome, Handgrip strength, Abdominal obesity, Body mass index, Cardiovascular disease

## Abstract

**Background:**

Adiposity is a major component of the metabolic syndrome (MetS), low muscle strength has also been identified as a risk factor for MetS and for cardiovascular disease. We describe the prevalence of MetS and evaluate the relationship between muscle strength, anthropometric measures of adiposity, and associations with the cluster of the components of MetS, in a middle-income country.

**Methods:**

MetS was defined by the International Diabetes Federation criteria. To assess the association between anthropometric variables (waist circumference (WC), waist-to-hip ratio (W/H), body mass index (BMI)), strength (handgrip/kg bodyweight (HGS/BW)) and the cluster of MetS, we created a MetS score. For each alteration (high triglycerides, low HDLc, dysglycemia, or high blood pressure) one point was conferred. To evaluate the association an index of fat:muscle and MetS score, participants were divided into 9 groups based on combinations of sex-specific tertiles of WC and HGS/BW.

**Results:**

The overall prevalence of MetS in the 5,026 participants (64% women; mean age 51.2 years) was 42%. Lower HGS/BW, and higher WC, BMI, and W/H were associated with a higher MetS score. Amongst the 9 HGS/BW:WC groups, participants in the lowest tertile of HGS/BW and the highest tertile of WC had a higher MetS score (OR = 4.69 in women and OR = 8.25 in men;p < 0.01) compared to those in the highest tertile of HGS/BW and in the lowest tertile of WC.

**Conclusion:**

WC was the principal risk factor for a high MetS score and an inverse association between HGS/BW and MetS score was found. Combining these anthropometric measures improved the prediction of metabolic alterations over either alone.

**Supplementary Information:**

The online version contains supplementary material available at 10.1186/s12933-021-01256-z.

## Background

Metabolic syndrome (MetS) is associated with a higher risk of cardiovascular disease (CVD) mortality and total mortality [1]. The cluster of the metabolic alterations that comprise MetS includes dysglycemia, low HDL-c, increased triglycerides, and elevated blood pressure. The accumulation of adiposity, predominantly in visceral tissue, is the cornerstone feature of the development of MetS [2, 3] and the growing prevalence of obesity is considered a principal determinant of the increased prevalence of MetS, type 2 diabetes mellitus and CVD globally [4–7]. Indeed, in the Latin American population, abdominal obesity has the highest population attributable risk for a first acute myocardial infarction [8].

The Prospective Urban Rural Epidemiological (PURE) study, an epidemiological cohort study with more than 150.000 participants worldwide showed that lower muscle strength evaluated with handgrip dynamometry, an index of overall strength in the general population, is also independently associated with CVD and cardiovascular mortality [9, 10]. The combination of obesity and low muscle strength is associated with an additive cardiovascular risk in high income countries [11], but there is a paucity of information in low-medium income countries. The present study aimed firstly to establish the prevalence of MetS within Colombia, a middle-income country, using the PURE database. We also aimed to determine the association between anthropometric indicators of adiposity, handgrip strength (HGS/BW), and the combination of the two and presence of alterations in the components of the cluster of metabolic alterations of MetS.

## Methods

### Population and study design

The PURE study design, coordinated by the Population Health Research Institute (PHRI Hamilton, ON, Canada), was described previously [12]. In the case of Colombia, the protocol was approved by the Fundación Cardiovascular de Colombia ethics committee. Participants were selected from both urban and rural communities from eleven departments across the country, allowing the collection of data from a sample that represents 51.29% of the population. A three-phase survey was applied, in which the first and second phases consisted of selecting the communities involved, and the third phase of selecting the homes included within those communities. A community was defined as the geographical area where a group of people with common characteristics lived. We considered a home rural if it was located more than 50 km from an urban center. A home was selected if a family member was between the ages of 35–70 years old and if the individuals intended to stay in this household for the next 4 years. Trained personnel made three attempts to contact a member of each household for door-to-door collection of information. We included all participants who completed and signed written consent.

### Data collection and risk factors

For each consenting participant, sociodemographic characteristics and cardiovascular risk factors were obtained. Blood pressure, anthropometrics and handgrip strength were also measured. A 10-mL fasting venous blood sample (≥ 8 h without any consumption of food or drinks) was obtained for the determination of various biochemical parameters in the central clinic lab, including lipid profile and glucose. Triglycerides, total cholesterol and high-density lipoprotein cholesterol were estimated by enzymatic colorimetric method in an automatic analyzer (Hitachi 917, Boehringer Mannheim) and LDL-c was calculated. For detecting dysglycemia, the enzymatic hexokinase method was applied to determine glucose levels in each sample. Age was categorized as < 50 years versus ≥ 50 years. Educational level was categorized as a high/medium level of education for those with a high school degree, university diploma or technical diploma. Individuals with a low educational level were those without schooling, primary schooling, or unknown academic history. We considered smokers all those who consumed a daily tobacco product in the last 12 months and included those who reported having quit smoking in the last year. Never drinking was defined as self-reported abstinence, former drinking was defined as having ceased alcohol consumption for 1 year or more, and current drinking was defined as consumption of alcohol in the past year.

MetS was defined according to the International Diabetes Federation (IDF) criteria [13], with at least three of the following factors: (i) central obesity (as documented by an abdominal circumference > 90 cm in men and > 80 cm in women, which are relevant ethnicity-specific thresholds), (ii) elevated triglyceride levels > 150 mg/dl (1.7 mmol/L) or having medical treatment for this lipid abnormality; (iii) reduced HDL- c levels < 40 mg/dl (1.0 mmol/L) in men and < 50 mg/dl (1.3 mmol/L) in women, or under specific medical treatment for this lipid abnormality including fibrates (PPAR alpha agonists) or statins; (iv) increased blood pressure (systolic blood pressure ≥ 130 mmHg or diastolic pressure ≥ 85 mmHg), including those on antihypertensive medication, or with a previous diagnosis of hypertension; (v) elevated fasting glucose levels (≥ 100 mg/dl). Trained research assistants used a sphygmomanometer (Omron® HEM-757) with a 14 × 48 cm cuff to measure blood pressure. Blood pressure was taken with no smoking, physical activity, or food consumption during the previous 30 min and after the participant sat for 5 min. Anthropometric measurements were taken following the standardized protocol of the PURE study. Weight was measured using a digital scale with the participant lightly clothed with no shoes. Height was measured to the nearest millimeter using a tape measure with the participant standing without shoes. Waist and hip circumferences were measured unclothed using a tape measure. The WC was considered the smallest circumference between the costal margin and the iliac crest. The hip circumference was measured at the level of the greater trochanters. BMI was calculated as the participant’s weight in kilograms divided by the square of the height in meters. Central adiposity was represented by W/H and was calculated dividing waist circumference by hip circumference. Participants were categorized by tertiles according BMI and W/H.

Handgrip strength was measured was evaluated on the individual's non-dominant hand using a Jamar dynamometer (Sammons Preston, Bolingbrook, IL, USA), according to a standardized protocol [9]. Standing, the participant held the dynamometer at the side of the body with the elbow flexed at 90-degree angle and was asked to squeeze the device as hard as possible for 3 s. This was repeated twice with 30 s rest between each attempt. Handgrip strength (kg) was divided by bodyweight (kg) to calculate HGS/BW in order to account for body mass, a key determinant of HGS. Participants were categorized in sex-specific tertiles according to both handgrip strength and HGS/BW. Physical activity (PA) was evaluated using the International Physical Activity Questionnaire (IPAQ). IPAQ which assesses physical activity undertaken across a comprehensive set of domains, including leisure-time physical activity, domestic and gardening activities, work-related physical activity, transport-related physical activity. Those who reported less than 600 MET-min/week were considered as having a low PA level, between 600 and 3000 MET-min/week were moderate, and a report quantified as more than 3000 MET-min/week were considered as having a high PA level [14]. These thresholds take into account that the IPAQ queries PA in multiple domains of daily life, resulting in higher median MET-minutes estimates than would be that estimated from considering leisure-time participation alone.

To assess the association between anthropometric variables (WC, W/H, BMI) and muscle strength (HGS/BW) and the cluster of MetS alterations, we developed a MetS score. One point was conferred for each alteration of the cluster of MetS as defined by IDF (elevated triglycerides, low HDL-c, dysglycemia, or high blood pressure), generating a score of 0 to 4 for each participant, a high score was considered if 2 or more points were achieved. WC was not included in the calculation of our metabolic score as it was also an outcome variable. As the analysis aimed to evaluate the interaction between central obesity/muscle strength and its relationship with MetS, the exclusion of WC avoids a circular argument whereby participants MetS score was influenced by abdominal perimeter.

### Statistical analysis

Descriptive statistics were computed for variables of interests and included absolute and relative frequencies of categorical factors. Testing for differences in categorical variables was accomplished using the Chi-square test. Moreover, we used unconditional multivariate logistic regression models to assess the associations between anthropometric variables and handgrip strength, and the MetS score. These analyses were adjusted for potential confounders, such as age, socioeconomic status, income and education level. We re-coded the anthropometric variables and handgrip strength into sex-specific tertiles and compared the risk of a higher MetS score in each tertile with the lowest category of risk (reference group). Odds ratio (OR) along with the 95% confidence interval (95% CI) were reported.

All statistical analysis was carried out using the R software version 3.6.2 (R Foundation for Statistical Computing). A P < 0.05 was considered statistically significant.

## Results

In total, 5026 participants were included in this analysis, of which 64% were women. The mean age was 51.2 ± 9.6 years and 51.5% of participants were older than 50 years. The overall prevalence of MetS was 42.1% (95% CI 40.7–43.5). Baseline characteristics of individuals with (n = 2116) and without MetS (n = 2910) are presented in Table 1. MetS was more frequent in women, people older than 50 years; it was also more frequent in individuals living in urban areas, former drinkers, and smokers. The prevalence of MetS was higher in participants with a lower level of education compared with those with a high school or college degree. The percentage of subjects with MetS was lower in tertile 1 of BMI (14.5%) compared to subjects in tertile 2 (56.2%) and tertile 3 (78%) (p < 0.001). In the lowest tertile of W/H, 14.9% met the MetS criterion, compared with subjects in 42.8% of those in tertile 2 and 67.8% of those in tertile 3 (p < 0.001). There were no significant differences in the prevalence of MetS across tertiles of HGS (tertile 3: 41.5%; tertile 2: 42.4%; tertile 1: 42.3%). However, the prevalence of MetS (25.8%) was significantly lower in the highest tertile of HGS/kg bodyweight (HGS/BW) compared to those in tertile 2 (32.4%) and tertile 1 (58%) (p < 0.001).

**Table 1:** Characteristics of participants with and without metabolic syndrome

Variables	Total	Without MetS (n %) N = 2910		With MetS (n %) N = 2116	
	(n)	Female	Male	Female	Male
Total	5026	1699 (33.8)	1211 (24.1)	1516 (30.2)	600 (11.9)
Age (years)					
< 50	2434	1013 (41.6)	604 (24.8)	572 (23.5)	245 (10.1)
> = 50	2592	686 (26.5)	607 (23.4)	944 (36.4)	355 (13.7)
Income (USD)					
High (> 700)	1563	558 (35.7)	318 (20.3)	445 (28.5)	242 (15.5)
Middle (350–700)	1720	567 (33)	438 (25.5)	505 (29.4)	210 (12.2)
Low (< 350)	1743	574 (32.9)	455 (26.1)	566 (32.5)	148 (8.5)
Education					
College/University	744	315 (42.3)	158 (21.2)	161 (21.6)	110 (14.8)
High school	974	384 (39.4)	195 (20)	281 (28.9)	114 (11.7)
None, primary, or unknown	3308	1000 (30.2)	858 (25.9)	1074 (32.5)	376 (11.4)
Location					
Rural	2789	831 (29.8)	808 (29)	826 (29.6)	324 (11.6)
Urban	2237	868 (38.8)	403 (18)	690 (30.8)	276 (12.3)
Alcohol use					
Never	2742	1213 (44.2)	316 (11.5)	1061 (38.7)	152 (5.5)
Before	789	180 (22.8)	248 (31.4)	201 (25.5)	160 (20.3)
Actual	1495	306 (20.5)	647 (43.3)	254 (17)	288 (19.3)
Tobacco use					
Never	3304	1318 (39.9)	531 (16.1)	1157 (35)	298 (9)
Before	1035	214 (20.7)	387 (37.4)	233 (22.5)	201 (19.4)
Actual	687	167 (24.3)	293 (42.6)	126 (18.3)	101 (14.7)
Physical activity					
Low	615	193 (31.4)	148 (24.1)	181 (29.4)	93 (15.1)
Moderate	1873	669 (35.7)	361 (19.3)	635 (33.9)	208 (11.1)
High	2538	837 (33)	702 (27.7)	700 (27.6)	299 (11.8)
BMI (kg/m2)					
Tertile 1	2160	1002 (46.4)	845 (39.1)	231 (10.7)	82 (3.8)
Tertile 2	1981	545 (27.5)	324 (16.4)	766 (38.7)	346 (17.5)
Tertile 3	885	152 (17.2)	42 (4.7)	519 (58.6)	172 (19.4)
Waist circumference (cm)					
Tertile 1	1696	1095 (64.6)	601 (35.4)	0 (0)	0 (0)
Tertile 2	1625	369 (22.7)	486 (29.9)	658 (40.5)	112 (6.9)
Tertile 3	1705	235 (13.8)	124 (7.3)	858 (50.3)	488 (28.6)
Waist/Hip ratio					
Tertile 1	1659	863 (52)	549 (33.1)	198 (11.9)	49 (3)
Tertile 2	1658	539 (32.5)	409 (24.7)	522 (31.5)	188 (11.3)
Tertile 3	1709	297 (17.4)	253 (14.8)	796 (46.6)	363 (21.2)
Handgrip strength (kg)					
Tertile 3	1655	601 (36.3)	366 (22.1)	477 (28.8)	211 (12.7)
Tertile 2	1595	509 (31.9)	409 (25.6)	471 (29.5)	206 (12.9)
Tertile 1	1776	589 (33.2)	436 (24.5)	568 (32.0)	183 (10.3)
Handgrip strength/Bodyweight					
Tertile 3	1705	756 (44.3)	508 (29.8)	333 (19.5)	108 (6.3)
Tertile 2	1662	555 (33.4)	402 (24.2)	510 (30.7)	195 (11.7)
Tertile 1	1659	388 (23.4)	301 (18.1)	673 (40.6)	297 (17.9)

MetS = Metabolic syndrome, BMI = body mass index, Waist/hip = Waist to hip ratio (W/H), HGS/BW = handgrip strength divided by bodyweight. T1 = Tertile 1, T2 = Tertile 2, T3 = Tertile 3

Reference values for each tertile of the anthropometric variables (F = female, M = male)

BMI (F): T1: 22.32 [15.82–25.00]; T2 27.29 [25.00–30.00]; T3 33.65 [30.01–58.37] BMI (M): T1 22.29 [16.22–24.98]; T2 27.18 [25.00–29.93]; T3 32.76 [30.00–58.03]. WC (F): T1 72.52 [42.00–79.00]; T2 83.89 [79.05–88.40]; T3 96.49 [88.50–143.80] WC (M): T1 76.66 [53.85–83]; T2 87.63 [83.05–92.00]; T3 100.24 [92.05–138.00]

W/H (F): T1 0.77 [0.42–0.82]; T2 0.85 [0.82–0.88]; T3 0.93 [0.88–2.22] W/H (M): T1 0.86 [0.53–0.90]; T2 0.93 [0.9–0.96]; T3 1.02 [0.96–2.38] Handgrip (F): T1 15.9 [7.70–19.30]; T2 21.5 [19.70–23.70]; T3 29.5 [24.0–83.3] Handgrip (M): T1 24.58 [8.00–30.0]; T2 34.03 [30.33–38]; T3 44.71 [38.3–90.0] HGS/BW (F): T1 0.24 [0.12–0.30]; T2 0.34 [0.30–0.39]; T3 0.48 [0.39–1.59] HGS/BW (M): T1 0.35 [0.11–0.43]; T2 0.49 [0.43–0.54]; T3 0.65 [0.54–1.76]

**Fig. 1:**
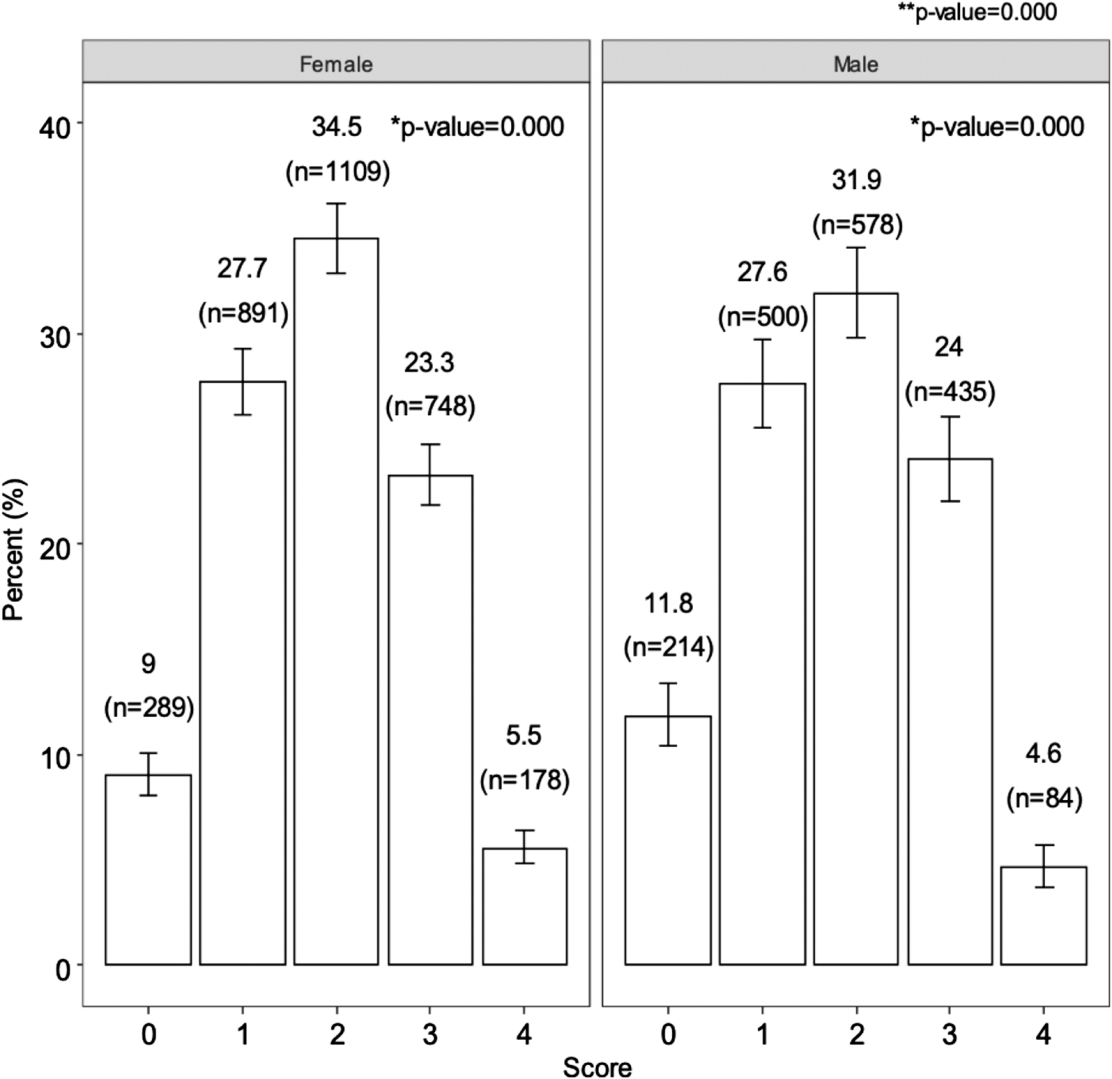
Percentage of females and males (CI 95%) in each metabolic score category. *p-value = Chi-square test within sex categories. ** p-value = Chi-square test for the association of score and test

Figure 1 shows the sex-specific distribution of the MetS scores. Approximately 10% of participants (9% women and 11.8% men) had no metabolic alterations, 5% had all the MetS score alterations (5.5% women and 4.6% men) and the majority had 2 alterations (women 34.5% and men 31.9%), this score being significantly more common than other scores (p < 0.001).

**Fig. 2:**
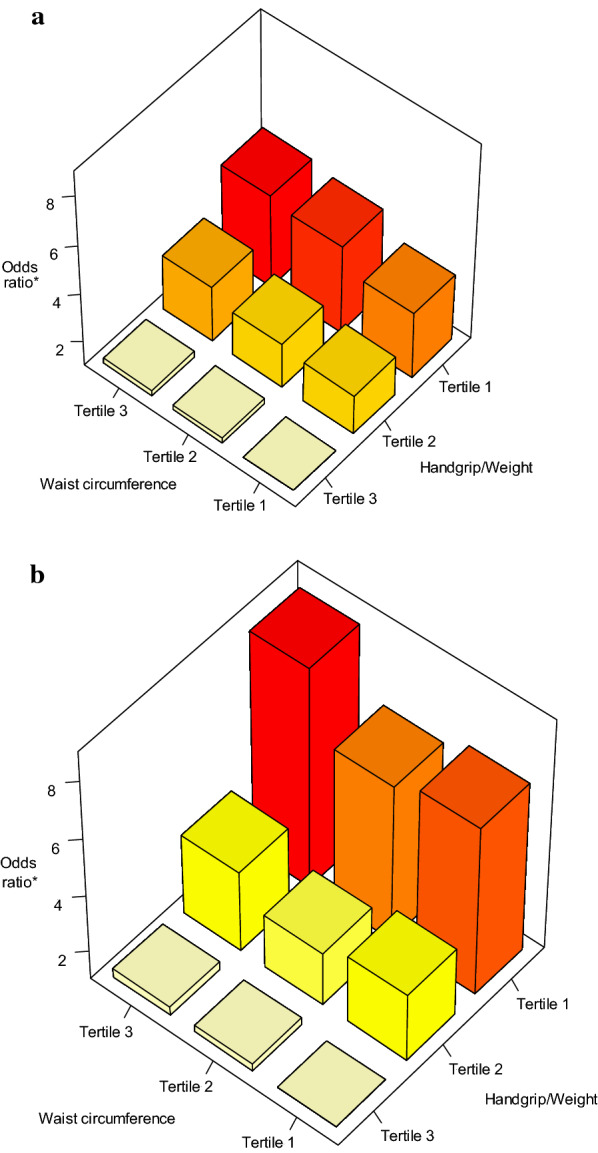
**a** Association between waist circumference and handgrip strength/bodyweight and the risk of higher MetS score in females. *Odds ratio obtained from main effects proportional odds model using waist circumference, handgrip strength /bodyweight and age associated with metabolic score. The reference categories are WC tertile 1 and HGS/BW tertile 3. **b** Association between waist circumference and handgrip strength/bodyweight and the risk of higher MetS score in males. *Odds ratio obtained from main effects proportional odds model using waist circumference, handgrip strength/bodyweight and age associated with metabolic score. The reference categories are WC tertile 1 and HGS/BW tertile 3

The association between anthropometric variables and the risk of a higher MetS score is shown in Table 2. A higher WC was associated with a risk of a higher MetS score, with women and men in the tertile 3 of WC (mean 96.4 cm and 100.2 cm, respectively) having a significantly higher risk of high score than those in tertile 1 after adjusting for covariates such as age, socioeconomic status, and education. This association was similar for W/H but less powerful compared to WC. Participants in tertile 3 of BMI (mean 33.6 kg/m^2^ in women and 32.7 kg/m^2^ in men) had a greater risk of a higher MetS score (OR = 3.20; CI 95%: 2.68–3.82 and OR = 4.65; CI 95% 3.51–6.15, respectively) compared to those in tertile 1 (mean 22.2 kg/m^2^). In women, lower HGS was associated with a significantly higher MetS score (T3 vs. T1 OR = 1.39 95% CI 1.20–1.61), although this association lost significance in the adjusted model. In men, there were no significant differences in MetS score across HGS tertiles. However, MetS score risk was significantly higher in women and men in the lowest tertiles of HGS/BW and remained significant after adjusting by covariates (OR = 1.91; 95% CI 1.63–2.24 and OR = 2.13; 95% CI 1.72–2.63 respectively). Figure 2a, b show the interaction between WC, HGS/BW and MetS score, adjusted by age. Those in WC tertile 3 and HGS/BW tertile 1 had more than fivefold risk compared to those in WC tertile 1 and HGS/BW tertile 3 (OR = 4.69; 95% CI 3.45–6.36 in women and OR = 8.25; 95% CI 5.38–12.64 in men). In both women and men, a summative effect is observed such that as across combinations of tertiles of WC and HGS/BW there was an incrementally increasing risk of a higher MetS score and was higher when combining WC and HGS/BW than over either alone (Additional file 1).

**Table 2:** Association between anthropometric variables and risk of higher MetS score

	Female					Male				
Anthropometric variables		Model 1^*^		Model 2^§^			Model 1^*^		Model 2^§^	
	Mean (Lwr-Upp)	OR	(95% CI)	OR	(95% CI)	Mean (Lwr-Upp)	OR	(95% CI)	OR	(95% CI)
*Handgrip strength (kg)*										
T3	29.5 (24–83.3)	Ref				44.7 (38.33–90)	Ref			
T2	21.5 (19.7–23.7)	1.28	1.10–1.50	1.12	0.95–1.31	34.0 (30.33–38)	0.78	0.64–0.96	0.74	0.60–0.91
T1	15.9 (7.7–19.3)	1.39	1.20–1.61	1.09	0.93–1.27	24.5 (8–30)	0.84	0.69–1.04	0.79	0.63–0.98
*Handgrip strength/bodyweight*										
T3	0.48 (0.39–1.59)	Ref				0.65 (0.54–1.76)	Ref			
T2	0.34 (0.3–0.39)	1.61	1.38–1.88	1.45	1.24–1.69	0.49 (0.43–0.54)	1.44	1.18–1.77	1.35	1.10–1.66
T1	0.24 (0.12–0.3)	2.32	1.99–2.71	1.91	1.63–2.24	0.35 (0.11–0.43)	2.33	1.89–2.86	2.13	1.72–2.63
*Waist circumference (cm)*										
T1	72.5 (42–79)	Ref				76.6 (53.85–83)	Ref			
T2	83.8 (79.0–88.4)	2.84	2.42–3.33	2.52	2.15–2.95	87.6 (83.05–92)	2.97	2.40–3.67	2.78	2.24–3.44
T3	96.4 (88.5–143.8)	4.72	4.02–5.55	3.98	3.39–4.69	100.2 (92.05–138)	6.93	5.55–8.65	6.38	5.07–8.02
*Waist to hip ratio*										
T1	0.77 (0.42–0.82)	Ref				0.86 (0.53–0.9)	Ref			
T2	0.85 (0.82–0.88)	2.36	2.02–2.77	2.13	1.81–2.49	0.93 (0.9–0.96)	2.08	1.69–2.56	2.08	1.69–2.56
T3	0.93 (0.88–2.2)	3.81	3.25–4.47	3.00	2.55–3.54	1.02 (0.96–2.38)	3.96	3.20–4.90	3.85	3.09–4.78
*Body mass index (kg/m*^*2*^*)*										
T1	22.3 (15.8–25)	Ref				22.2 (16.22–24.98)	Ref			
T2	27.2 (25–30)	2.29	1.98–2.64	2.15	1.85–2.48	27.1 (25–29.93)	3.39	2.81–4.09	3.33	2.74–4.03
T3	33.6 (30.0–58.3)	3.38	2.84–4.02	3.20	2.68–3.82	32.7 (30–58.03)	4.92	3.75–6.47	4.65	3.51–6.15

^*^Unadjusted analysis

^§^Analysis adjusted by age, socioeconomic status, income, education level,alcohol use, tabacco use, location, physical activity

## Discussion

### Prevalence of metabolic syndrome

The overall prevalence of MetS in this cohort of 5026 Colombian adults was 42.1%. A lower prevalence was reported by Higuita-Guitierrez in Colombian adults (of which 50.5% were under 65 years of age) attending a public chronic disease control program (35.4%) [15], while Barranco-Ruiz noted a prevalence of 54.9% in 1637 Colombians over 60-year-olds (mean age 70 ± 9 years) [16]. We observed double the prevalence of MetS in those over 50 years (> 50: 50.1%, < 50: 23.6%). Aging is associated with an increase in adipose tissue and a decreased muscle mass [17], body composition changes which predispose to the development of metabolic alterations. The prevalence of MetS was higher in women (47.1% vs. 33.1% in men), aligning with that previous reported in Latin American populations [18, 19]. Changes in hormonal status and estrogen/testosterone ratio during and after menopause likely contribute to the differences in the prevalence of age-related MetS in women [20]. Lower educational level was associated with a higher prevalence of MetS (43.9% vs. 36.4%). Educational level is an indicator of social inequity, lower levels reflecting not only less schooling, but also a higher risk of unhealthy life habits, and lower access to employment and physical activity participation. Social factors associated with MetS prevalence, should be further examined.

### Combining adiposity and muscle strength measurements increases the prediction of metabolic alterations.

We found that lower muscle strength and higher central adiposity as defined by waist circumference, were independently associated with a higher MetS score, representing a greater number of alterations of the components of the MetS cluster. We also noted that combining WC and HGS/BW, a potential proxy fat: muscle index, had a summative effect on the risk of having a higher MetS score.

Our cross-sectional analysis showed a stronger association between a higher MetS score and WC than BMI, confirming previous studies showing that in Latin-American and Chinese population, WC is a stronger predictor of major cardiovascular events such as myocardial infarction or stroke than BMI, particularly in men [8, 21]. Similarly, in diabetic Chinese adults, high visceral fat measured by a visceral adiposity index and WC were associated with a higher prevalence of diabetic kidney disease and CVD compared to BMI [22]. These findings may be related to the higher inflammatory load associated with visceral adipose tissue accumulation, and inflammation is considered a key factor associated with insulin resistance, MetS and CVD [23, 24]. The low-grade pro-inflammatory state characterized by high C-reactive protein levels is observed in adults and youth in our population with high visceral adiposity [25, 26]. However, the accumulation of visceral fat is not the only contributing factor in the development of a pro-inflammatory state. The accumulation of cardiac fat is also associated with higher levels of pro-inflammatory cytokines such as IL-6, IL-1, TNF-α, and the expression of adipokine fatty acid-binding protein 4 (FABP4) that are associated with the development of MetS and the extent of coronary artery disease [27, 28]. Hence, overall fat measurement should not be underestimated. For example, in a cohort of 1,332 Italian children and adolescents (14.4 ± 1.8 yrs), when comparing different adiposity indices and body composition, the diagnostic accuracy of BMI in identifying MetS was similar to that of WC, W/H, or body mass fat index (BMI x fat mass% by impedance x WC) [29]. However, BMI cannot discriminate between lean body mass and fat mass; hence, BMI is not necessarily an appropriate parameter of excessive adiposity. Body fat distribution may be more valuable than overall adiposity in the prediction of metabolic alterations. This aligns with the concept of an obesity paradox whereby subjects with higher BMI levels were shown to have lower levels of cardiovascular events [30]. Obesity induced alterations in body composition include both an increase in adipose and in low-density lean tissue, without an increment in normal- lean density tissue, suggesting a fatty infiltration of muscular tissue [31].

Furthermore, studies in Colombian adults have demonstrated that individuals with a high BMI due to higher muscle mass have a lower risk of CVD than individuals with the same BMI due to elevated adipose mass [32]. This highlights that not only adipose tissue influences insulin action, other tissues such as muscle and hepatic tissue also affect this interaction. Therefore, in our population, WC continues to be the most applicable, easy to perform anthropometric indicator of adiposity and predictor of metabolic alterations and CV risk. Furthermore, rather than a specific weight value, the cardiometabolic dysfunction produced by the adipose tissue's inflammation and its involvement in the muscle tissue should be managed.

Few studies have examined associations between strength, adiposity, and MetS or its components in adults in low and middle-income countries and considered its association with CVD and mortality [1]. A study in Colombian college students (n = 1795) found that both higher visceral adiposity and lower HGS/BW were associated with higher metabolic risk. However, the protective effect of muscle strength was seen in overweight/ obese but not normal-weight subjects [33]. We found that the protective effect of relative muscle strength was maintained across all tertiles of WC, which may relate to the larger sample, wider range of values, and lower mean HGS/BW in the present population. Moreover, low muscle mass and strength and increased adipose tissue is associated with higher total mortality (HR = 1.24; 95% CI 1.12–1.37, p < 0.001) [11].

### Low Relative HGS (HGS/BW) as a marker of insulin resistance, MetS, and CVD

The PURE study, a large international prospective cohort that included the present population, demonstrated an association between low HGS and CVD and all-cause mortality in the population as a whole [9]. In the present analysis, individuals in the lowest tertile of relative HGS (HGS/BW) had more than double the prevalence of MetS than those in the highest tertile, although this association lost significance when using absolute HGS (unadjusted for bodyweight). This aligns with the stronger association between HGS/BW than absolute handgrip strength and metabolic alterations reported in a Korean adult population [34]. HGS/BW is the common means to account for the influence of total body mass itself associated with muscle mass and strength increases.

In a sample of Chinese adults of similar size as the present study, and mean age of 46.7 years (SD 14.3), subjects in the highest quartile of HGS/BW had a better lipid profile and blood glucose levels compared to those in the lowest quartile [35]. Similar to our population, lower HGS/BW was significantly associated with a higher overall MetS score. Additionally, in a sample of 3350 subjects (mean age 59.2 ± 9.1 years) from the CHARLES study, representative of the Chinese population, Shen et al. [36] showed that compared to subjects in the highest quartile, those in quartile 1 to 3 had a progressively higher risk of MetS (Q3: OR 1.49 (0.95, 2.34), 1.67 (1.08, 2.59) and 1.76 (1.12, 2.78) in men, respectively), and (1.14 (0.82, 1.58), 1.30 (1.02, 1.57) and 1.28 (1.03, 1.55) in women respectively).

Relative strength, handgrip adjusted by bodyweight or BMI, is an appropriate marker of insulin resistance. A recent analysis of 2451 adults over 50 years old in the Korea National Health and Nutrition Examination Survey showed that relative HGS (adjusted for BMI) was negatively associated with HOMA-IR index in both men and women, − 0.141 and − 0.139, respectively (p < 0.001) [37], an effect maintained after adjusting for conventional risk factors. Furthermore, they compared various relative HGS measures, and found that the best MetS diagnostic performance was HGS/body fat mass (AUC males: 0.72 (0.66–0.77), females 0.61 (0.57–0.66)) followed by HGS/BW(males: 0.63 (0.57–0.69), females: 0.58 (0.53–0.63)) and HGS/BMI (males: 0.61 (0.55–0.67), females: 0.55 (0.50–0.60), respectively [38]. However, measures of body composition would be required to estimate fat mass, a measurement not widely available; therefore, HGS/BW represents a cost-effective and applicable measure from the clinical perspective with a diagnostic performance only slightly inferior to that of HGS:fat in the prediction metabolic alterations associated with insulin resistance.

Several levels of evidence support the notion that muscle strength is protective, and more so than muscle mass [39, 40]. Prospective studies have established that low muscle strength, typically characterized using handgrip dynamometry, is predictive of cardiometabolic risk and mortality, independent of aerobic fitness and physical activity [9, 41]. Epidemiological evidence demonstrates that participation in a relatively low frequency of resistance/strength training, a single session or less than 1 h a week, is protective against CV events and mortality independent of aerobic training [42]. Furthermore, intervention studies also consistently show benefits of strength training on components of MetS and other relevant markers of CVD risk, such as C-reactive protein [43]. Nonetheless, these interventions have almost exclusively been conducted in high-income countries, and further studies are needed to evaluate the impact of maintaining and/or increasing muscle strength in individuals with MetS within low and middle-income countries as part of efforts to achieve the goal of decreasing the burden of cardiovascular diseases as proposed by the United Nations [44]. This is particularly relevant in low and middle-income countries on the basis that in these regions (1) there are steeper increases in the burden of chronic disease in low and middle-income countries [45] (2) lower muscle strength is reported compared to high -income countries [9] and (3) the protective effect of muscle strength on cardiometabolic health may be accentuated in individuals with lower birth weight, an indicator or poorer early life nutrition and a more common phenotype in the lower socioeconomic status within middle-income countries [26]. Considering the association between MetS cluster metabolic alterations and CVD, our findings suggest that public health strategies should not only focus on adiposity but also identify and address lower muscular strength in our population [10, 46].

## Limitations

Our study has the limitation of cross-sectional analyses, in that we demonstrated associations between adiposity, strength, and MetS in our population without establishing causality in these associations. We did not use body composition methods such as bioimpedance or dual-energy X-ray absorptiometry that estimate muscle and fat mass.

## Conclusion

Lower HGS/BW and higher WC are independently and additively association with the presence of alterations in the components of the MetS cluster. Therefore, quantifying relative muscle strength in an individual through the simple, quick and low-cost measurement of handgrip dynamometry in addition to the classic anthropometric measurements of adiposity (i.e. WC and W/H), could be a useful screening strategy to improve the identification of individuals at high risk of MetS and CVD. Having greater muscle strength could be a protective factor against the metabolic alterations that constitute this syndrome. Handgrip strength is also associated with frailty and other non-cardiometabolic related chronic physical and mental health outcomes [47], so from a clinical perspective it can also contribute to the wider a screening of patient health. There is a need in low and middle-income countries for greater attention to the development of studies that evaluate the function and protective characteristics of muscle mass/strength on CVD and other cardiometabolic risk factors. Our results provide further evidence indicating the need to establish public health strategies to identify, prevent and “treat” low muscle mass and function in a community context.

## Supplementary Information

**Additional file 1.** Association between waist circumference and handgrip strength/bodyweight, the risk of higher MetS score.

## Data Availability

The datasets used and/or analyzed during the current study are available from the corresponding author on reasonable request.

## Abbreviations

BMI: Body mass index
CVD: Cardiovascular disease
HGS/BW: Handgrip/kg bodyweight
IDF: International Diabetes Federation
MetS: Metabolic syndrome
OR: Odds ratio
PA: Physical activity
PURE: The Prospective Urban Rural Epidemiological study
WC: Waist circumference
W/H: Waist-to-hip ratio

## References

[1] Mottillo S, Filion KB, Genest J, Joseph L, Pilote L, Poirier P (2010). The metabolic syndrome and cardiovascular risk a systematic review and meta-analysis. J Am Coll Cardiol.

[2] Haczeyni F, Bell-Anderson KS, Farrell GC (2018). Causes and mechanisms of adipocyte enlargement and adipose expansion. Obes Rev.

[3] Vu JD, Vu JB, Pio JR, Malik S, Franklin SS, Chen RS (2005). Impact of C-reactive protein on the likelihood of peripheral arterial disease in United States adults with the metabolic syndrome, diabetes mellitus, and preexisting cardiovascular disease. Am J Cardiol.

[4] Collaborators GBDO, Afshin A, Forouzanfar MH, Reitsma MB, Sur P, Estep K (2017). Health effects of overweight and obesity in 195 countries over 25 years. N Engl J Med.

[5] Aguilar M, Bhuket T, Torres S, Liu B, Wong RJ (2015). Prevalence of the metabolic syndrome in the United States, 2003–2012. JAMA.

[6] Raposo L, Severo M, Barros H, Santos AC (2017). The prevalence of the metabolic syndrome in Portugal: the PORMETS study. BMC Public Health.

[7] Ansarimoghaddam A, Adineh HA, Zareban I, Iranpour S, HosseinZadeh A, Kh F (2018). Prevalence of metabolic syndrome in Middle-East countries: Meta-analysis of cross-sectional studies. Diabetes Metab Syndr.

[8] Lanas F, Avezum A, Bautista LE, Diaz R, Luna M, Islam S (2007). Risk factors for acute myocardial infarction in Latin America: the INTERHEART Latin American study. Circulation.

[9] Leong DP, Teo KK, Rangarajan S, Lopez-Jaramillo P, Avezum A, Orlandini A (2015). Prognostic value of grip strength: findings from the Prospective Urban Rural Epidemiology (PURE) study. Lancet.

[10] Yusuf S, Joseph P, Rangarajan S, Islam S, Mente A, Hystad P (2020). Modifiable risk factors, cardiovascular disease, and mortality in 155 722 individuals from 21 high-income, middle-income, and low-income countries (PURE): a prospective cohort study. Lancet.

[11] Tian S, Xu Y (2016). Association of sarcopenic obesity with the risk of all-cause mortality: a meta-analysis of prospective cohort studies. Geriatr Gerontol Int.

[12] Teo K, Chow CK, Vaz M, Rangarajan S, Yusuf S, Group PI-W. The Prospective Urban Rural Epidemiology (PURE) study: examining the impact of societal influences on chronic noncommunicable diseases in low-, middle-, and high-income countries. Am Heart J. 2009;158(1):1–7 e1. 10.1016/j.ahj.2009.04.019 19540385

[13] Alberti KG, Zimmet P, Shaw J, Group IDFETFC (2005). The metabolic syndrome–a new worldwide definition. Lancet.

[14] IPAQ. Guidelines for data processing and analysis of the International Physical Activity Questionnaire (IPAQ)-Short and Long Forms 2005. https://sites.google.com/site/theipaq/

[15] Higuita-Gutierrez LF, Martinez Quiroz WJ, Cardona-Arias JA (2020). Prevalence of Metabolic Syndrome and Its Association with Sociodemographic Characteristics in Participants of a Public Chronic Disease Control Program in Medellin, Colombia, in 2018. Diabetes Metab Syndr Obes.

[16] Barranco-Ruiz Y, Villa-Gonzalez E, Venegas-Sanabria LC, Chavarro-Carvajal DA, Cano-Gutierrez CA, Izquierdo M (2020). Metabolic syndrome and its associated factors in older adults: a secondary analysis of SABE Colombia in 2015. Metab Syndr Relat Disord.

[17] Tieland M, Trouwborst I, Clark BC (2018). Skeletal muscle performance and ageing. J Cachexia Sarcopenia Muscle.

[18] Wong-McClure RA, Gregg EW, Barcelo A, Lee K, Abarca-Gomez L, Sanabria-Lopez L (2015). Prevalence of metabolic syndrome in Central America: a cross-sectional population-based study. Rev Panam Salud Publica.

[19] Marquez-Sandoval F, Macedo-Ojeda G, Viramontes-Horner D, Fernandez Ballart JD, Salas Salvado J, Vizmanos B (2011). The prevalence of metabolic syndrome in Latin America: a systematic review. Public Health Nutr.

[20] Pucci G, Alcidi R, Tap L, Battista F, Mattace-Raso F, Schillaci G (2017). Sex- and gender-related prevalence, cardiovascular risk and therapeutic approach in metabolic syndrome: a review of the literature. Pharmacol Res.

[21] Xing Z, Peng Z, Wang X, Zhu Z, Pei J, Hu X (2020). Waist circumference is associated with major adverse cardiovascular events in male but not female patients with type-2 diabetes mellitus. Cardiovasc Diabetol.

[22] Wan H, Wang Y, Xiang Q, Fang S, Chen Y, Chen C (2020). Associations between abdominal obesity indices and diabetic complications: Chinese visceral adiposity index and neck circumference. Cardiovasc Diabetol.

[23] Garcia RG, Perez M, Maas R, Schwedhelm E, Boger RH, Lopez-Jaramillo P (2007). Plasma concentrations of asymmetric dimethylarginine (ADMA) in metabolic syndrome. Int J Cardiol.

[24] Ridker PM, Hennekens CH, Buring JE, Rifai N (2000). C-reactive protein and other markers of inflammation in the prediction of cardiovascular disease in women. N Engl J Med.

[25] Bautista LE, Lopez-Jaramillo P, Vera LM, Casas JP, Otero AP, Guaracao AI (2001). Is C-reactive protein an independent risk factor for essential hypertension?. J Hypertens.

[26] Gomez-Arbelaez D, Camacho PA, Cohen DD, Saavedra-Cortes S, Lopez-Lopez C, Lopez-Jaramillo P (2016). Neck circumference as a predictor of metabolic syndrome, insulin resistance and low-grade systemic inflammation in children: the ACFIES study. BMC Pediatr.

[27] Gormez S, Erdim R, Akan G, Caynak B, Duran C, Gunay D (2020). Relationships between visceral/subcutaneous adipose tissue FABP4 expression and coronary atherosclerosis in patients with metabolic syndrome. Cardiovasc Pathol..

[28] Kralisch S, Fasshauer M (2013). Adipocyte fatty acid binding protein: a novel adipokine involved in the pathogenesis of metabolic and vascular disease?. Diabetologia.

[29] Radetti G, Fanolla A, Grugni G, Lupi F, Sartorio A (2019). Indexes of adiposity and body composition in the prediction of metabolic syndrome in obese children and adolescents: which is the best?. Nutr Metab Cardiovasc Dis.

[30] Park SJ, Ha KH, Kim DJ (2020). Body mass index and cardiovascular outcomes in patients with acute coronary syndrome by diabetes status: the obesity paradox in a Korean national cohort study. Cardiovasc Diabetol.

[31] Kelley DE, Slasky BS, Janosky J (1991). Skeletal muscle density: effects of obesity and non-insulin-dependent diabetes mellitus. Am J Clin Nutr.

[32] Ramirez-Velez R, Correa-Bautista JE, Lobelo F, Izquierdo M, Alonso-Martinez A, Rodriguez-Rodriguez F (2016). High muscular fitness has a powerful protective cardiometabolic effect in adults: influence of weight status. BMC Public Health.

[33] Garcia-Hermoso A, Tordecilla-Sanders A, Correa-Bautista JE, Peterson MD, Izquierdo M, Prieto-Benavides D (2019). Handgrip strength attenuates the adverse effects of overweight on cardiometabolic risk factors among collegiate students but not in individuals with higher fat levels. Sci Rep.

[34] Chun SW, Kim W, Choi KH (2019). Comparison between grip strength and grip strength divided by body weight in their relationship with metabolic syndrome and quality of life in the elderly. PLoS ONE.

[35] Li D, Guo G, Xia L, Yang X, Zhang B, Liu F (2018). Relative handgrip strength is inversely associated with metabolic profile and metabolic disease in the general population in China. Front Physiol.

[36] Shen C, Lu J, Xu Z, Xu Y, Yang Y (2020). Association between handgrip strength and the risk of new-onset metabolic syndrome: a population-based cohort study. BMJ Open..

[37] Kim YM, Kim S, Bae J, Kim SH, Won YJ (2021). Association between relative hand-grip strength and chronic cardiometabolic and musculoskeletal diseases in Koreans: a cross-sectional study. Arch Gerontol Geriatr..

[38] Song P, Zhang Y, Wang Y, Han P, Fu L, Chen X (2020). Clinical relevance of different handgrip strength indexes and metabolic syndrome in Chinese community-dwelling elderly individuals. Arch Gerontol Geriatr..

[39] Newman AB, Kupelian V, Visser M, Simonsick EM, Goodpaster BH, Kritchevsky SB (2006). Strength, but not muscle mass, is associated with mortality in the health, aging and body composition study cohort. J Gerontol A Biol Sci Med Sci.

[40] Visser M, Goodpaster BH, Kritchevsky SB, Newman AB, Nevitt M, Rubin SM (2005). Muscle mass, muscle strength, and muscle fat infiltration as predictors of incident mobility limitations in well-functioning older persons. J Gerontol A Biol Sci Med Sci.

[41] Kim Y, White T, Wijndaele K, Westgate K, Sharp SJ, Helge JW (2018). The combination of cardiorespiratory fitness and muscle strength, and mortality risk. Eur J Epidemiol.

[42] Liu Y, Lee DC, Li Y, Zhu W, Zhang R, Sui X (2019). Associations of resistance exercise with cardiovascular disease morbidity and mortality. Med Sci Sports Exerc.

[43] Saeidifard F, Medina-Inojosa JR, West CP, Olson TP, Somers VK, Bonikowske AR (2019). The association of resistance training with mortality: A systematic review and meta-analysis. Eur J Prev Cardiol.

[44] United Nations. United Nations Sustainable Development Internet 2017. https://www.un.org/sustainabledevelopment/health/

[45] Global Burden of Disease Study C. Global, regional, and national incidence, prevalence, and years lived with disability for 301 acute and chronic diseases and injuries in 188 countries, 1990–2013: a systematic analysis for the Global Burden of Disease Study 2013. Lancet. 2015;386(9995):743–800. 10.1016/S0140-6736(15)60692-4 PMC4561509 26063472

[46] Otero J, Cohen DD, Herrera VM, Camacho PA, Bernal O, Lopez-Jaramillo P. Sociodemographic factors related to handgrip strength in children and adolescents in a middle income country: The SALUS study. Am J Hum Biol. 2017;29(1). 10.1002/ajhb.22896 27427286

[47] Rijk JM, Roos PR, Deckx L, van den Akker M, Buntinx F (2016). Prognostic value of handgrip strength in people aged 60 years and older: a systematic review and meta-analysis. Geriatr Gerontol Int.

