# Supplementary material for: The prediction of Metabolic Syndrome alterations is improved by combining waist circumference and handgrip strength measurements compared to either alone
Source: Cardiovasc Diabetol. 2021 Mar 22;20:68. doi: 10.1186/s12933-021-01256-z (PMC7986558; doi:10.1186/s12933-021-01256-z)
Supplement: Supplementary file 1 — Additional file 1. Association between waist circumference and handgrip strength/bodyweight, the risk of higher MetS score. [file 12933_2021_1256_MOESM1_ESM.docx]

**Additional file 1.** Association between waist circumference and handgrip strength/bodyweight, the risk of higher MetS score.

|  | **Female**§ | **Male**§ |
| --- | --- | --- |
| WC T1 - HGS/BW T3 | Reference | Reference |
| WC T1 - HGS/BW T2 | 1.25 (0.89-1.76) | 1.25 (0.78-2.01) |
| WC T1 - HGS/BW T1 | 1.25 (0.81-1.92) | 1.4 (0.81-2.41) |
| WC T2 - HGS/BW T3 | 2.55 (1.81-3.59) | 3.19 (2.04-4.98) |
| WC T2 - HGS/BW T2 | 2.73 (1.96-3.8) | 2.75 (1.78-4.27) |
| WC T2 - HGS/BW T1 | 3.21 (2.26-4.55) | 3.79 (2.34-6.13) |
| WC T3 - HGS/BW T3 | 3.78 (2.48-5.76) | 6.69 (3.75-11.93) |
| WC T3 - HGS/BW T2 | 4.49 (3.17-6.36) | 6.32 (3.96-10.1) |
| WC T3 - HGS/BW T1 | 4.69 (3.45-6.36) | 8.25 (5.38-12.64) |
| WC = Waist Circumference, HGS/BW = handgrip strength divided by bodyweight. T1= Tertile 1, T2= Tertile 2, T3= Tertile 3.  Reference values for each tertile of the anthropometric variables (F= female, M=male)  WC (F): T1 72.52 [42.00-79.00]; T2 83.89 [79.05-88.40]; T3 96.49 [88.50-143.80] WC (M): T1 76.66 [53.85-83]; T2 87.63 [83.05-92.00]; T3 100.24 [92.05-138.00]  HGS/BW (F): T1 0.24 [0.12-0.30]; T2 0.34 [0.30-0.39]; T3 0.48 [0.39-1.59] HGS/BW (M): T1 0.35 [0.11-0.43]; T2 0.49 [0.43-0.54]; T3 0.65 [0.54-1.76]  § Analysis adjusted by age, socioeconomic status, income, education level,alcohol use, tabacco use, location, physical activity. | | |
